# Chinese Herbal Medicine in Ameliorating Diabetic Kidney Disease via Activating Autophagy

**DOI:** 10.1155/2019/9030893

**Published:** 2019-11-16

**Authors:** Yuyang Wang, Hailing Zhao, Qian Wang, Xuefeng Zhou, Xiaoguang Lu, Tongtong Liu, Yongli Zhan, Ping Li

**Affiliations:** ^1^Department of Nephrology, Guang'anmen Hospital, China Academy of Chinese Medical Sciences, Beijing 100053, China; ^2^Tianjin University of Traditional Chinese Medicine, Tianjin 301617, China; ^3^Beijing Key Laboratory for Immune-Mediated Inflammatory Diseases, Institute of Clinical Medical Sciences, China-Japan Friendship Hospital, Beijing 100029, China; ^4^Beijing University of Chinese Medicine, Beijing 10029, China

## Abstract

Diabetic kidney disease (DKD), a leading cause of end-stage renal disease (ESRD), has become a serious public health problem worldwide and lacks effective therapies due to its complex pathogenesis. Recent studies suggested defective autophagy involved in the pathogenesis and progression of DKD. Chinese herbal medicine, as an emerging option for the treatment of DKD, could improve diabetic kidney injury by activating autophagy. In this review, we briefly summarize underlying mechanisms of autophagy dysregulation in DKD, including AMP-activated protein kinase (AMPK), the mechanistic target of rapamycin (mTOR), and the sirtuin (Sirt) pathways, and we particularly concentrate on the current status of Chinese herbal medicine treating DKD by regulating autophagy. The advances in our understanding regarding the treatment of DKD via regulating autophagy with Chinese herbal medicine will enhance the clinical application of Chinese medicine as well as discovery of novel therapeutic agents for diabetic patients.

## 1. Introduction

Diabetic kidney disease (DKD) covers a broad spectrum of kidney abnormalities, ranging from glomerular mesangial matrix deposition, thickening of the basement membrane, podocyte foot process effacement, and renal tubular hypertrophy to interstitial fibrosis and glomerular sclerosis [1]. This debilitating disorder is a widely recognized major microvascular complication of diabetes, affecting almost 35-40% of patients with both type 1 and type 2 diabetes [2, 3]. According to data from the international diabetes federation, the worldwide prevalence of diabetes continues to increase rapidly, and the number of people with diabetes around world will rise to 692 million in 2045 if nothing is done [4]. Meanwhile, DKD represents a leading cause of end-stage renal disease (ESRD) among western societies, occurs in approximately 50% of patients with diagnosed diabetes worldwide [1, 5]. Although DKD is an important component of diabetes microvascular injury and cardiovascular risk factors, a satisfactory explanation of pathogenesis is still unavailable at the moment [6, 7].

Autophagy is a newly identified cellular process that has been inferred in the pathogenesis of DKD. It is generally considered that autophagy is an important self-defensive mechanism for cell survival and protects cells against stress responses like oxidative stress, hypoxic stress, DNA damage, and ER stress [8]. Activated autophagy can occur when renal cells are exposed to a large variety of conditions, including nutrient/energy deficiency, hypoxia, ROS accumulation, protein aggregation, organelle damage, or intracellular pathogen invasion. While the relationship between autophagy and DKD was not fully understood yet, emerging evidence obtained from laboratory and clinical patients shows defective autophagy associated with DKD [9–12]. Reduced autophagy is closely related to pathological alterations in DKD such as albuminuria, glomerular hyperfiltration, and mesangial broadening. Furthermore, autophagy inhibition with bafilomycin A1, beclin-1 siRNA, or autophagy-related gene 5 (Atg5) knockout results in accelerated ROS accumulation [13] and glomerulosclerosis [14]. In contrast, activating autophagy with rapamycin, sequestosome1 knockout can attenuate fibrosis and DKD progression.

In China, traditional Chinese herbal medicine acts as an alternative approach in the treatment of DKD, which is widely used in clinical practice. Current strategies to treat DKD recommend multifactorial management including blood pressure control with renin-angiotensin system inhibitors and hypoglycemic agents like insulin, dipeptidyl peptidase-4 (DPP-4) inhibitors, sodium glucose cotransporter type 2 (SGLT2) inhibitors, glucagon-like peptide 1 (GLP1), lipid control, and dietary restrictions [15]. Despite those strategies showing renoprotective effects against DKD, the incidence of ESRD attributed to DKD is still high. Numerous Chinese herbal formulas have been confirmed by clinical evidence in the treatment of DKD [16, 17], and active ingredients like Tripterygium glycoside [18], resveratrol [19], and berberine [20] could also improve DKD supported by accumulating researches. Recent studies reveal Chinese herbal medicine can induce autophagy [12, 18, 19], which may provide an explanation for its renoprotective action in DKD therapy.

Increasing attention has begun to focus on the role of autophagy in the progression of DKD and renoprotective effects of herbal medicine in triggering autophagy. This review discusses the recent advances of Chinese herbal medicine in treating DKD via activating autophagy, with emphasis on the core mechanisms associated with autophagy induction.

## 2. Autophagy

**Figure 1:**
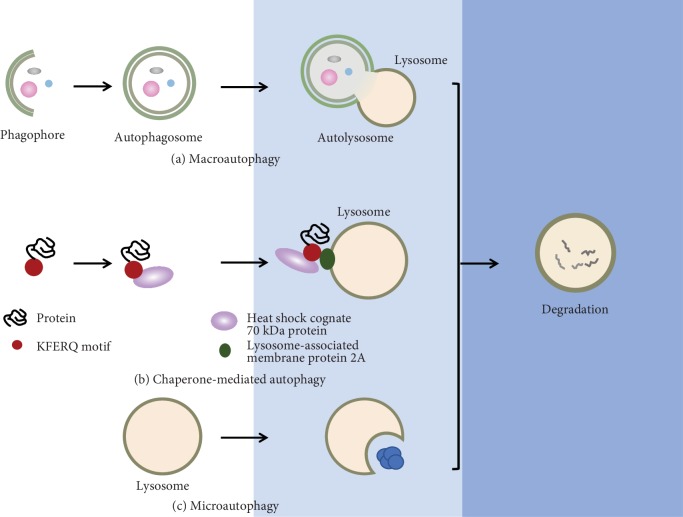
Three types of autophagy. (a) Macroautophagy is characterized by the appearance of the double membrane-bound vesicles called autophagosome, which aids in the transport of cytoplasmic constituents and organelles, and finally degrading the entrapped cytosolic components by fusing with lysosome. (b) Chaperone-mediated autophagy entails selective translocation of target proteins across the lysosome membrane into the lysosome lumen via a process requiring chaperone. Cytosolic proteins carrying the KFERQ-like pentapeptide motif are recognized by chaperone heat shock cognate 70 kDa protein (HSC70) and subsequent associate with the lysosome-associated membrane protein 2A (LAMP2A), facilitating its translocation. (c) Microautophagy, a poorly understood process, involves the direct engulfment of cytosolic contents with the lysosome membrane.

Autophagy, also called self-eating, refers to a lysosome-dependent cellular process involved in the clearance, degradation, and recycling of cytoplasmic target material, which is highly conserved from yeast to eukaryotic cells [21]. By transporting long-lived proteins and damaged organelles to lysosomes for degradation, autophagy is vital in maintaining cellular homeostasis during a range of physiological and pathological situations, including energy restriction or various pathological stresses like hypoxia [8, 22]. It is generally accepted that three forms of autophagy, macroautophagy, microautophagy, and chaperone-mediated autophagy, are occurring in mammalian cells (Figure 1). Macroautophagy, commonly known as autophagy, has been extensively studied and the focus of this review.

The major steps of autophagy are composed of initiation, elongation, maturation, fusion with the lysosomes, and degradation [23]. It is initiated by the formation of an isolation membrane or a phagophore containing portions of the cytoplasm. The origin of this double-membrane vesicle is controversial and may rise from either the plasma membrane, rough endoplasmic reticulum, or the Golgi. ULK complex, including ULK1, family interacting protein of 200 kDa (FIP200), autophagy-related protein 13 (Atg13), and Atg101, mediates the initiation process, followed by recruiting beclin1, vacuolar protein sorting 15 (Vps15), autophagy-related protein 14 (Atg14), and class III phosphatidylinositol-3-kinase (PI3K) to provide a platform for the formation of autophagosomes [21, 23]. Two ubiquitin-like conjugation systems, ATG12–ATG5–ATG16L complex and the microtubule-associated protein light chain 3-phosphatidyl ethanolamine (MAPLC3/LC3-PE), are required in the elongation and closure of autophagosome [24]. LC3II can be detected in autophagosome membranes before autophagosome fuses with the lysosome, which has been recognized as a marker of autophagy. In the autophagosome–lysosome fusion stage, closed autophagosome combines with lysosomes to form autolysosomes, a process regulated by vesicle-associated membrane protein8 (VAMP8), lysosome membrane protein2 (LAMP2), Rab7, and syntaxin17 [21]. Once autolysosomes occur, the contents of autophagosome are degraded by lysosome hydrolases and then released into cytoplasm for recycle, thereby completing the autophagy process.

## 3. Impaired Autophagy and Its Mechanisms in DKD

Recent studies have found that protein accumulation caused by the imbalance of protein synthesis and degradation in diabetic kidneys exacerbates glomerular hypertrophy and further renal pathological injury, which contributes to the occurrence and development of DKD [25]. Autophagy serves as a crucial pathway in protein degradation, although its relationship with DKD remains to be elucidated; increasing evidence suggested that defective autophagy arisen from the diabetic condition may contribute to the occurrence and development of DKD. Renal biopsy specimens from patients with type 2 diabetes displayed deficient autophagy in proximal tubules, as shown by the accumulation of p62/SQSTM1. Meanwhile, a marked reduction of serum beclin1 has been reported among DKD patients [9, 26]. Data obtained from in vivo animal experiments in ob/ob diabetic mice [10], db/db mice [11], and streptozotocin- (STZ-) induced diabetic rats [12] further demonstrated that renal autophagy activity was significantly downregulated under diabetic environment. However, stimulating autophagy via calorie restriction mitigated glomerular and tubulointerstitial pathologies in DKD mice [10]. In addition, autophagy has been proven to play a critical role in maintaining the integrity and function of renal intrinsic cells [24] and protecting cells against various intracellular stress.

**Figure 2:**
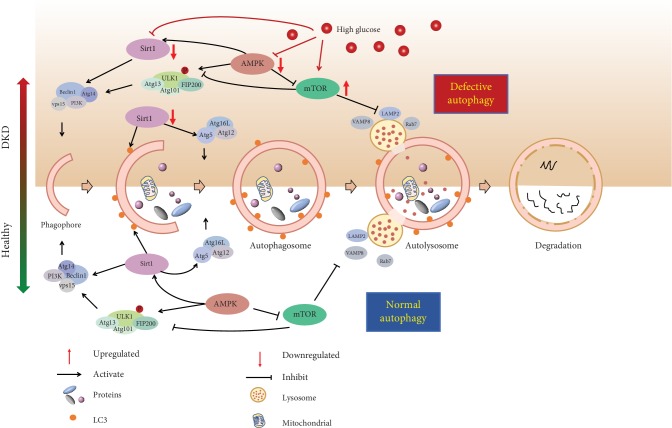
The process and regulatory mechanisms of autophagy during healthy conditions and DKD. The process of autophagy involves five steps: formation of phagophore, phagophore elongation, appearance of autophagosome, formation of autolysosome, and degradation. Under normal conditions, activity of autophagy is kept at a higher level in order to maintain cellular homeostasis via the clearance, degradation, and recycling of cytoplasmic long-lived proteins and damaged organelles. Under diabetic conditions, high glucose leads to a marked suppression of autophagy activity mainly through nutrient-sensing pathways including mTOR, AMPK, and Sirt1, resulting in aberrant protein degradation and may contribute to the pathogenesis of DKD.

The best-studied regulatory mechanisms of autophagy have been described in energy status signaling; thus nutrient-sensing pathways including AMP-activated protein kinase (AMPK), the mechanistic target of rapamycin (mTOR), and the sirtuins (Sirt) are recognized to mediate autophagy in mammalian cells [8, 24]. Recent studies have identified dysregulation of these pathways impairs autophagy activity and may contribute to the exacerbated renal injury in DKD (Figure 2).

### 3.1. mTOR

mTOR is a serine/threonine protein kinase that serves as a critical regulator of cellular nutrient levels and receives a large amount of attention during DKD. Two distinct functional complexes, termed the mTOR complex 1 (mTORC1) and mTOR complex 2 (mTORC2), have been identified [22]. mTORC1 is widely studied which consists of mTOR, regulatory-associated protein of mTOR (RAPTOR), G protein *β*-subunit-like protein (G*β*L), proline-rich Akt substrate of 40 kDa (PRAS40), and DEP domain protein that interacts with mTOR (DEPTOR). Tuberous sclerosis complex (TSC), the most prominent upstream negative regulator of mTORC1, can be activated by protein kinase B (Akt) and extracellular signal-regulated kinase (ERK) in response to changes of environmental conditions and sequentially inhibits autophagy [27]. Activation of mTORC1 directly brings about the inhibition of downstream substrate Unc-51 like autophagy-activating kinase (ULK1) and thereby reducing autophagy. Furthermore, under amino acid-stimulated conditions, mTORC1 is recruited to the lysosomal membrane and involved in the lysosome biogenesis, which leads to impaired autophagy [22]. Recent studies have suggested that the defective autophagy associated with increased mTOR activity may be implicated in the occurrence and progression of DKD. Elevated mTOR activity is observed in db/db mice, STZ-induced diabetic rats, and patients with DKD [28–30]. Podocyte-specific mTORC1 activation in mice by knocking out TSC1 results in significant proteinuria and glomerulopathy under both diabetic and nondiabetic conditions, paralleled by glomerular hypertrophy; mesangial expansion; and decreased number of podocytes and phenotypic change [31]. Moreover, further study indicated that inhibition of mTORC1 activity by rapamycin treatment could attenuate apoptosis and fibrosis of kidney cortexes in db/db mice by restoring autophagy [28]. These data suggest the crucial role of mTOR in the pathogenesis of DKD as well as therapeutical targets.

### 3.2. AMPK

AMPK is a heterotrimeric kinase broadly expressed in eukaryotes and composed of three subunits, *α*, *β*, and *γ*. By sensing the changing AMP/ATP ratio in the cell, AMPK serves as an essential mediator of the cellular response to energy-depleted conditions and maintaining energy homeostasis [32]. Upon stimulation, AMPK directly phosphorylates ULK1 or crosstalk with mTORC1, resulting in autophagy induction [21]. AMPK is believed to positively regulate autophagy and has been found to be downregulated in DKD. Decreased autophagy in db/db mice and STZ-induced rats resulted from suppression of AMPK activity and was inferred in the pathogenesis of DKD [11, 33]. In response to high glucose, AMPK reduced autophagy activity in podocyte, HK-2, and human glomerular endothelial cells (HGECs) with increased cytotoxicity and apoptosis [11, 20, 33]. However, administration of AICAR, an AMPK agonist, can reverse high glucose-induced podocyte injury [34], implying the therapeutical potential of activated autophagy in regard to DKD.

### 3.3. Sirt1

Sirtuins, identified as NAD+-dependent type III deacetylase, are essential in the regulation of intracellular metabolic and redox status. There are seven members of the mammalian Sirtuins (Sirt1-Sirt7), among which Sirt1 is the most studied and has been implied to play an important role in the pathogenesis of metabolic diseases like DKD [27]. Once activated, Sirt1 enhances autophagy via deacetylating crucial autophagic proteins Atg5, Atg7, and LC3 in a nicotinamide adenine dinucleotide (NAD+)-dependent way. Furthermore, Sirt1 can interact with AMPK and mTOR, leading to hyperactivation of autophagy. A study demonstrated that Sirt1 was activated by AMPK in the modulation of energy metabolism and further deacetylates the downstream targets such as transcription factor Forkhead box O3 (FOXO3) [35]. In addition to the link with AMPK, Sirt1 inhibits the activity of mTOR through phosphorylating TSC2 [27]. As a potent positive regulator of autophagy, the expression of renal Sirt1 was suppressed in a range of diabetic animal models including STZ-treated mice, db/db mice, and Wistar fatty (fa/fa) rats [36]. Genetically engineered diabetic animals with marked remission of Sirt1 in widespread, podocyte-specific, and renal tubular-specific showed aggravation of albuminuria and glomerular abnormalities, whereas overexpression of Sirt1 or treatment with BF175 has been reported to display a preventive effect against diabetic-associated podocyte damage and the development of DKD [37]. Moreover, the reduced Sirt1 expression was observed in the glomeruli of DKD patients [38].

Other molecules, like class III PI3K complex (PI3K), Forkhead box protein O3 (FOXO3), and transcription factor EB (TFEB), are also described in the process of autophagy during DKD [39], which indicates the potential of autophagy to become an emerging drug target in the treatment of DKD. Notably, clinical trials have been conducted to confirm that some drugs with autophagy-inducing potential are effective in DKD, such as sirolimus [40] and resveratrol [41]. However, none of their effects has been explained through inducing autophagy yet, implying more novel agents targeted on activating autophagy needs to be further explored in laboratory and clinical trials.

## 4. Mechanisms of Chinese Herbal Medicine Treating DKD via Activating Autophagy

With the support of a unique and integrated theoretical system, herbal medications have traditionally been used for relieving clinical symptoms associated with DKD and improving renal function for centuries in Chinese medical practice and some Asian countries. Clinical evidence obtained from China has confirmed the beneficial effect of Chinese herbal medicine in the treatment of DKD [42, 43], including Tangshen formula [16], Liu Wei Di Huang pill and Ginkgo Biloba Tablets [17], Qidan Dihuang Grain [43], silymarin [44], and *Tripterygium Hook. f.* extracts [45]. Besides, a population-based cohort study by Chen et al. revealed that Chinese herbal remedies lowered ERSD and mortality rates among DKD patients [46]. Recent studies on DKD suggest certain Chinese herbal agents exert autophagy-inducing functions [18], implying autophagy activation may act as a potential target for its renoprotective action in DKD therapy; meanwhile, the mechanisms of how herbal medicine exerts renal benefits on DKD are addressed through different pathways related to autophagy mainly involving mTOR, AMPK, and Sirt1 (Table 1).

### 4.1. Chinese Herbal Medicine Treating DKD via Regulating mTOR-Mediated Autophagy

Triptolide, principally active constituents of an ancient Chinese herb *Tripterygium Hook. f.*, has been demonstrated to display protective effects on renal fibrosis and autophagy in DKD animal model [47]. Triptolide decreased the expression of profibrogenic factors involving fibronectin and collagen IV, accompanied with restoration in the activities of the miR-141-3p/PTEN/Akt/mTOR pathway [47]. Such an improvement of triptolide on fibrosis can be relieved by the application of 3-methyladenine and Atg5 siRNA, which further validated that autophagy was indispensable for triptolide in alleviating renal fibrosis.

Mangiferin is a natural xanthone component derived from many plants such as *Anemarrhena asphodeloides Bge*. Evidence has been obtained from the STZ-induced diabetic rat model that mangiferin could delay the process of DKD and protect podocytes [12]. Twelve-week administration of mangiferin during DKD development could decrease podocyte loss and increase the expression of podocyte marker nephrin in diabetic kidney [12]. Besides, mangiferin treatment also enhanced the expression of LC3 II and suppressed the storage of p62, accompanied with certain autophagic positive regulators like AMPK was upregulated, while autophagic negative factors such as mTOR downregulated.

Hispidulin is a flavonoid component isolated from *Plantago asiatica L.* Importantly, hispidulin has been illustrated to protect podocyte in high glucose-cultured conditions against apoptosis by regulating Pim1-p21-mTOR signaling axis-induced autophagy [48]. Inhibition of Pim1 mitigated the increase of autophagic factors LC3 II, beclin1, and restraint of p62 by hispidulin under high-glucose stress, suggesting that the autophagy-inducing effect of hispidulin was dependent on Pim1. Besides, treatment of hispidulin in podocytes reduced the phosphorylated mTOR and ULK1, and combination with rapamycin hardly showed coordinative effects for activating autophagy.

### 4.2. Chinese Herbal Medicine Treating DKD via Regulating AMPK-Mediated Autophagy

Astragaloside IV, a saponin compound from *Astragalus membranaceus* Bge., has exhibited many biological activities in renal protection [49]. Administration of Astragaloside IV for 8 consecutive weeks in STZ-induced diabetic mice resulted in remission of albuminuria and glomerulosclerosis, paralleled by inhibition in podocyte apoptosis and restoration in impaired autophagy [50]. Blockade of autophagy or AMPK activation could neutralize an Astragaloside IV-exhibited favorable effect, suggesting that Astragaloside IV attenuated the progression of DKD in part by AMPK-mediated autophagy induction.

Berberine, an extract from *Rhizoma Coptidis* and *Phellodendron amurense Rupr*, is commonly used in Chinese medicine for its pharmacological effects such as hypoglycemic, hypolipidemic, and anti-inflammation, which has drawn increasing attention for treating hepatic steatosis, hyperlipidemia, and diabetes [20, 51]. Importantly, berberine can stimulate autophagy through activating AMPK and ameliorate high glucose-induced podocyte apoptosis [20]. However, autophagic inhibitor, 3-methyladenine or bafilomycin A1, significantly mitigates the protective effect of berberine on podocyte apoptosis.

Cyclocarya paliurus belongs to traditional Chinese herbal plants and enriches triterpenic acids. It has been reported that Cyclocarya paliurus triterpenic acids mitigated microalbumin excretion and renal fibrosis in STZ-induced diabetic rats via increasing autophagy, along with the downregulation of mTOR and the upregulation of AMPK [33]. Furthermore, treatment of Cyclocarya paliurus triterpenic acids alleviated high glucose-induced defective autophagy and hyperactivation of apoptosis in HK-2 through regulating the AMPK/mTOR pathway. The beneficial effects of Cyclocarya paliurus triterpenic acids can be reversed by AMPK inhibitor compound C, suggesting that the effect of which on ameliorating renal function was achieved by AMPK-mediated autophagy.

### 4.3. Chinese Herbal Medicine Treating DKD via Regulating Sirt1-Mediated Autophagy

Resveratrol is a polyphenol found in *Reynoutria japonica Houtt.* and grapes; some beneficial effects in DKD have been observed in db/db mice [19, 52] and STZ-induced diabetic rats [53] via promoting autophagy. Evidence from a clinical trial has shown that resveratrol could decrease albumin/creatinine ratio in DKD patients compared with placebo, accompanied by increased serum antioxidant enzymes like superoxide diamutase, catalase, and reduced malondialdehyde [41]. Resveratrol has been considered as Sirt1 activators, a study on DKD rats further revealed that resveratrol could alleviate renal function abnormalities and enhance autophagy by increasing Sirt1 activity [53]. In addition, autophagy inducing of resveratrol in db/db mice and podocytes in vitro appeared to protect kidney tissues against apoptosis and attenuate DKD-associated renal impairment by suppressing miR-383-5p and upregulating miR-18a-5p [19, 52]. Moreover, the restraining effects of resveratrol on podocyte apoptosis could be reversed by autophagy inhibitor 3-methyladenine and Atg5 short hairpin RNA (shRNA), while reinforced by autophagy activator rapamycin.

Azuki bean is a common dietary plant widely found in east Asian. A recent study revealed that azuki bean extracts could downregulate plasma glutathione levels and block expression of heme oxygenase 1, superoxide dismutase 1, and p47phox protein in DKD experimental rats [54], in line with the ameliorated renal dysfunction and disturbance of glucose metabolism. Further mechanism study indicated that the protective effects of azuki bean extracts were partially associated with Sirt1-mediated autophagy induction.

Astragaloside IV has also been reported to play a crucial role in decreasing ECM protein overproduction and triggering autophagy via regulating Sirt1/NF-*κ*B signaling in KK-ay mice [55]. Astragaloside IV treatment could repress the expression of key fibrosis marker in high glucose-induced mesangial cells. However, administration of 3-methyladenine eradicated the ameliorative role of Astragaloside IV and enhanced mesangial cell activation.

Abelmoschus manihot, an extract of *Abelmoschus manihot medic*, has been widely used in Chinese medicine to treat a variety of kidney diseases including DKD. In type 2 diabetic rats, Abelmoschus manihot has shown renoprotective effects in improving podocyte decrease and alleviating the pathology changes in the kidney, along with the increased Sirt1 expression and upregulated autophagic factors like LC3B, Atg5, and Atg12, suggesting that the effects of Abelmoschus manihot are likely related to autophagy stimulation [56].

### 4.4. Chinese Herbal Medicine Treating DKD via Regulating Other Pathway-Mediated Autophagy

Tangshen formula (TSF), a developed proprietary compound prescription, composed of seven natural herbs involving astragalus, burning bush, rehmannia, bitter orange, cornus, rhubarb, and notoginseng. Our laboratory has been working on revealing the efficacy of TSF in the treatment of DKD as well as its potential mechanism [16]. A multicenter double-blinded randomized placebo-controlled trial performed by our team validated the additional benefits of the TSF compared with placebo in attenuating deterioration of 24-hour urinary protein and eGFR decrease among DKD patients with macroalbuminuria; meanwhile, further investigations found that TSF exhibited therapeutical potential on renal fibrosis by downregulating the TGF-*β*/Smad3 signaling pathway in streptozotocin-treated rats [57]. Lately, we revealed that TSF could suppress ECM deposition and collagen III protein expression in db/db mice and NRK52E cell exposed to high glucose [58], in line with increased autophagic markers LC3 II and accumulated p62, implying that the effects may be partially associated with promyelocytic leukemia zinc finger protein-mediated autophagy activation. In addition, Astragali radix served as the monarch component of TSF, and its main active extract Astragaloside IV is able to prevent renal fibrosis in the diabetic KK-Ay mice through autophagy activation [55]. Similar upregulation of autophagy was further confirmed in STZ-induced mice after the intervention of Astragaloside IV, accompanied with marked remission in albuminuria and glomerulosclerosis.

Ferulic acid, one of the common dietary polyphenols, could markedly decrease serum Cr, BUN levels, and urinary albumin/urinary creatinine ratio in STZ-exposed rats, along with rehabilitation of excessive inflammation via reducing the expression of inflammatory parameters and inhibiting the NF-*κ*B-mediated pathway [59, 60]. Further study has shown ferulic acid enhances autophagy-related proteins beclin1 and LC3-II expressions and sequentially alleviates oxidative damage. Moreover, low autophagy activity has been observed in high glucose-cultured NRK52E cells [59]; pharmaceutical inhibitors of autophagy counteracted the beneficial effect of ferulic acid in autophagy induction and cell death.

Celastrol is a triterpenoid and isolated from *Tripterygium Hook. f.* As a NF-*κ*B inhibitor, celastrol has been shown to significantly reduce serum Cr levels and urinary albumin excretion, as well as improve lipid abnormalities and histologic alterations in db/db mouse kidney through modulating inflammatory processes [61]. In vitro experiment shows consistent results in high glucose-exposed podocytes [62], Celastrol is reported to protect podocyte against inflammatory response and restore podocyte viability via heme oxygenase 1-mediated autophagy activating.

Tripterygium glycoside, an extract from a natural herb *Tripterygium Hook. f.* with strong immunosuppressive and anti-inflammatory effects, has been reported to decrease proteinuria and improve renal dysfunction in DKD through numerous randomized controlled clinical trials [45, 63]. In high-glucose serum from db/db mouse induced podocytes, tripterygium glycoside intervention significantly decreased apoptotic activity, along with elevated LC3-II/LC3-I ratios and reduced expression of p62 [18]. Silencing beta-arrestin-1 enhanced the autophagic levels and ameliorated podocyte injury, suggesting that the renal benefit of tripterygium glycoside is achieved partially by increasing autophagy.

Curcumin, derived from *Curcuma Longa L.*, has been implicated in treating DKD. Intervention of curcumin in diabetic patients [64] and experimental rats [65] resulted in a significant decrease in diabetes-related renal damage. AGE overload gradually induces apoptosis in tubular epithelial cells. Wei et al. [66] demonstrated that curcumin reduced apoptosis activity and upregulated protective autophagy by phosphorylating the PI3K/Akt signaling pathway in AGE-induced rat tubular epithelial cells. Inhibition of autophagy by 3-methyladenine reversed its effect on cell apoptosis, further confirming the protective role of curcumin via inducing autophagy.

## 5. Barriers and Future Perspectives

Advances in traditional herbal medicine suggest Chinese herbal remedies can be considered promising agents in the prevention and treatment of DKD. However, the clinical application of Chinese herbal medications targeted on stimulating autophagy is pretty limited, yet due to the following concerns and barriers, meanwhile, the future directions may be included but not limited. (1) Increasing experimental evidence reveals an essential role for induced autophagy of herbal medications in the process of DKD, but these researches mostly focus on the observation of autophagy only; detailed mechanistic studies to identify the direct drug targets are rare. To aim at this barrier, the role of herbal medicine on DKD should be examined using high-quality animal studies, such as genetically engineered diabetic animals. (2) The potential side effects associated with herbal medicine may be underrecognized and underreported partly due to lacking data from long-term follow-up of patients. Some Chinese herbal agents with autophagy-inducing potential such as Tripterygium wilfordii may cause unfavorable side effects like liver toxicity, infertility, and hematopoietic disorders [67]. Increasing attention should be given to the side effects related to Chinese herbal medicine and improvement of the reporting system for adverse events before herbal medications can emerge as a viable remedy in the treatment of DKD. (3) Although most studies in Chinese herbal medicine support a kidney-protective effect of activating autophagy on DKD, a few discoveries suggest otherwise points. Findings from recent studies demonstrated that some herbal extracts may exert beneficial effects on DKD through inhibiting autophagy. Paeoniflorin could inhibit AGE-mediated autophagy inducing and protect mesangial cells from collagen IV accumulation [68], and ginsenoside-Rg1 maintained redox homeostasis of podocyte and NRK52E cell by suppressing autophagy [69, 70]. Despite that these studies are limited to in vitro experiments, which also reminds us to explain the action of hyperactivated autophagy on DKD with caution, further investigations in laboratory are required to interpret the role of autophagy in the process of DKD.

## 6. Conclusion

Although current treatment measures can effectively delay the progress of DKD, the incidence is still increasing worldwide. In this review, we have mainly discussed the role of Chinese herbal medicine in treating DKD and the underlying mechanisms that contribute to autophagy activation. The commonly used active compounds extracted from Chinese herbs, such as tripterygium glycoside, Abelmoschus manihot, resveratrol, Astragaloside IV, ferulic acid, are promising candidates for preventing and treating DKD, which are closely related to activating autophagy. We believe that further mechanism studies of Chinese herbal medicine regulating autophagy will be beneficial to the clinical treatment and provide new therapeutic strategies for DKD.

## Figures and Tables

**Table 1:** Chinese herbal medicine treating DKD by activating autophagy.

Names	Models	Targeted pathways	Ref.
Triptolide	STZ+HFD-induced rats and HG-induced HMCs	miR-141-3p/PTEN/Akt/mTOR pathway	[47]
Mangiferin	STZ-induced rats; HG-induced podocytes	AMPK/mTOR	[12]
Hispidulin	HG-induced podocytes	Pim1/p21/mTOR	[48]
Astragaloside IV	STZ-induced mice; HG-incubated podocytes	AMPK; Sirt1/NF-*κ*B	[50, 55]
Berberine	HG-induced podocytes	AMPK	[20]
Cyclocarya paliurus triterpenic acids	STZ-induced rats; HG-induced HK-2	AMPK/mTOR	[33]
Resveratrol	Db/db mice; STZ-induced rats; HG-induced podocytes; hypoxia-induced NRK52E	Sirt1; miRNA-18a-5p; miR-383-5p	[19, 52, 53]
Azuki bean extract	STZ-induced rats	Sirt1	[54]
Abelmoschus manihot	UN+HFD+STZ-induced mice	Sirt1	[56]
TSF	Db/db mice; HG-induced NRK52E	PLZF	[58]
Ferulic acid	STZ-induced rats; HG-induced NRK52E	Beclin1 and LC3-II	[59]
Celastrol	HG-induced podocytes	HO-1	[62]
Tripterygium glycoside	Db/db mice serum induced podocytes	*β* arrestin1	[18]
Curcumin	AGE-induced NRK52E	PI3K/Akt	[66]

Abbreviations: STZ: streptozotocin; HFD: high-fat diet; HG: high glucose; HMCs: human mesangial cells; PTEN: phosphatase and tensin homolog; Akt: AKT serine/threonine kinase 1; mTOR: mammalian target of rapamycin; AMPK: AMP-activated protein kinases; Pim1: Pim-1 protooncogene; Sirt1: sirtuin 1; NF-*κ*B: nuclear factor-kappa b; HK-2: human renal proximal tubular cell; NRK52E: rat renal proximal tubular cell; UN: unilateral nephrectomy; TSF: Tangshen formula; PLZF: promyelocytic leukemia zinc finnger protein; LC3-II: microtubule-associated protein 1-light chain 3; HO-1: heme oxygenase 1; RAGE: receptor for advanced glycation end products; PI3K: phosphatidylinositol 3-kinases.
